# GAMEPLANS: A template for robust digital evidence strategy development

**DOI:** 10.1111/1556-4029.15655

**Published:** 2024-11-07

**Authors:** Graeme Horsman

**Affiliations:** ^1^ Cranfield University Bedford UK

**Keywords:** digital evidence, digital evidence strategy, digital forensics, digital investigation, police

## Abstract

Law enforcement officers should now expect to encounter forms of digital evidence at most of their inquiries, and as a result ensure they are prepared to effectively deal with it. This should involve the production of a digital evidence strategy (DES) which describes those actions required of any investigative team to effectively identify, collect, examine, and evaluate any digital devices/data, while also defining the circumstances for when it is appropriate to conduct such tasks. To help officers to produce robust DESs this work provides a DES template which utilizes the “GAMEPLANS” acrostic to identify nine fundamental components that are required of all DESs—“*G*”–*Grounds for investigation*; “*A*”*–Authorization*; “*M*”*–Method of investigation*; “*E*”*–Evaluation of the meaning of any findings*; “*P*”*–Proportionality*; “*L*”*–Logic*; “*A*”*–Agreement*; “*N*”–*Necessity*; “*S*”–*Scrutiny*. Each of these components are described including the sub‐tasks that are contained within each, which any officer constructing a robust and effective DES must address (and provide evidence of having addressed). To support this, a DES template file is also provided, which can be utilized by officers.

**Highlights**

- A discussion of the importance of digital evidence strategies is offered.
- The GAMEPLANS digital evidence strategy template is proposed.
- Nine fundamental components of digital evidence strategies are explained.

## 1 INTRODUCTION

The National Police Chiefs' Council [1] note that “today, virtually every crime has a digital element, often involving vast amounts of complex data, and this presents policing with a serious challenge,” a view that is widely shared [2, 3, 4]. The ability to effectively examine the contents of digital devices and the data they produce and store is now a crucial part of any investigation and effective criminal justice system [5], yet undertaking this task is far from straightforward and not the only challenge facing investigators when dealing with digital devices. Whilst it is acknowledged that the forensic examination of devices and their data can itself be a complicated task [6], there are also difficulties involved with understanding and identifying the value that any source of digital data may have in regard to an inquiry, and establishing an appropriate and effective course of investigative conduct for dealing with it [7, 8]. For this reason, when any inquiry is either expected to have, or is confirmed to maintain a “digital element” (i.e., digital devices considered to contain information that may be of relevance to an inquiry) which may require further investigation, a digital evidence strategy (DES) should be produced and followed [9, 10, 11].

A DES should describe those actions required of any investigative team (which may include front line staff, forensic experts and managers) to effectively identify, collect, examine, and evaluate any digital devices/data, whilst also defining the circumstances when it is appropriate to conduct such tasks [7, 8]. A “good” DES should serve as a good practice guide by outlining the behaviors and activities that are required in order to conduct a thorough investigation of any case's circumstances where digital devices and/or data are expected to be present. This should increase the chance of any inquiry‐relevant data being correctly identified and processed, of which any results can then be used to inform future investigative and legal decision making. Conversely, a poor DES (or the absence of one) may lead to missed opportunities, flawed decision making or the deployment of digital forensic (DF) practices which could compromise any investigation or fail to effectively address any requirements of the case. Such concerns have already been raised via His Majesty's Inspectorate of Constabulary and Fire & Rescue Services [12] report into the use of DF by law enforcement which raised questions regarding how it is deployed in England and Wales.

Whilst it is acknowledged that “investigators must be able to identify, prioritize, and manage” investigative opportunities arising from digital evidence at crime scenes [12], a lack of training (or training budgets), resources, and skills can mean this task may not always be conducted effectively. The National Police Chiefs' Council's [13] vision statement for policing in 2030 recognizes the need to develop “individual leaders to be more data literate and digitally competent,” and whilst greater awareness of the value of digital devices to an investigation is required [14], achieving it will need significant investment. This is acknowledged by Walcott [15] who states that “the challenge is getting policing into a position where it can access, process and examine such large volumes of data in a way that meets the standards we expect from criminal investigations.” A failure to upskill officers in this area will arguably have a detrimental impact on law enforcement's ability to provide swift and reliable justice. Digital illiteracy and methods that are ineffective for risk assessing and prioritizing digital devices for examination will likely reduce the speed of police inquiries, and may lead to tensions amongst both police officers [16] and likely the wider public. Investigative teams can no longer approach their case work unprepared to tackle digital evidence and instead expect to have to deal with it in most of their work. As a result, it is vital that they maintain and abide by appropriate strategies for doing this effectively.

This work aims to support law enforcement staff when investigating crime where digital devices and their data may be involved through the provision of a DES template. This template utilizes the “GAMEPLANS” acrostic to identify nine components that are required of all DESs – “*G*”–*Grounds for investigation*; “*A*”–*Authorization*; “*M*”–*Method of investigation*; “*E*”–*Evaluation of the meaning of any findings*; “*P*”–*Proportionality*; “*L*”–*Logic*; “*A*”–*Agreement*; “*N*”–*Necessity*; “*S*”–*Scrutiny*. Each of these components are described including the sub‐tasks that are contained within each, which any officer constructing a robust and effective DES must address (and provide evidence which shows how they have addressed them). To support this, a DES template file is also provided, which can be utilized by officers.

## 2 DESs AND THEIR IMPORTANCE

There is increasing recognition of the need for frontline police to improve their knowledge and awareness of digital evidence and the forensic capability within their force [17]. This is echoed by His Majesty's Inspectorate of Constabulary and Fire and Rescue Services [12] who state that whilst “the use of crime scene managers (CSMs) and investigative strategies to support investigators is commonplace,” it has been observed that there is a lack of knowledge and skills underpinning the requirements for handling and processing digital evidence. To ensure that our police service is effective when investigating crime, there is a need to help officers make effective investigative decisions when digital evidence is involved, which includes determining what to take from a scene, and how to examine it [14]. Concerns exist that at present, such decisions may not be being made effectively where work by Thompson and Manning [18] found that “samples from two years of serious crime investigations established that 50% of enquiries missed all digital investigative opportunities. Where a digital opportunity was identified, potential subsequent digital enquiries were missed 47% of the time.” The failure to identify and capture relevant information is a fundamental error [19] and there is a need to help police officers to better prepare and plan for any digital opportunities which may arise or be present at any given incident.

Whilst His Majesty's Inspectorate of Constabulary and Fire and Rescue Services [12] have suggested “police forces are overwhelmed and ineffective” when it comes to DF, the challenge for police when dealing with digital evidence is far wider than concerns surrounding its forensic services. Officers must have the knowledge, skills, and awareness to identify any potential value that digital devices and their data may offer their inquiry, and, when such digital opportunities are spotted, the necessary processes and procedures required to capitalize upon them. In essence, officers must now expect and plan for the presence of digital evidence in their inquiries where a DES is often required which must wrap around the entire investigative process to support those who follow it at all stages of their inquiry. A well‐constructed DES should help to raise awareness of the digital opportunities that may exist within a scene, preventing them from being missed. It should also stop the unnecessary seizure of exhibits which add to existing backlogs which are causing delays to justice [20]; something that is currently a recognized problem [12, 21]. A DES should also define appropriate investigative conduct and the circumstances where it can and should be deployed. For this reason, DESs play a pivotal role during investigations by mapping out acceptable investigative behavior, and are an important tool for those conducting lawful inquiries, if they are constructed properly. To help create robust DESs, this work proposes the GAMEPLANS DES template. The GAMEPLANS structure takes into account and incorporates key positions and best practices outlined in DF research, policy, and best practice narratives, often with a focus on how such investigations are conducted in England and Wales. However, it is suggested that many of the components of the GAMEPLANS structure may be transferable into other jurisdictions.

## 3 GAMEPLANS

**FIGURE 1:**
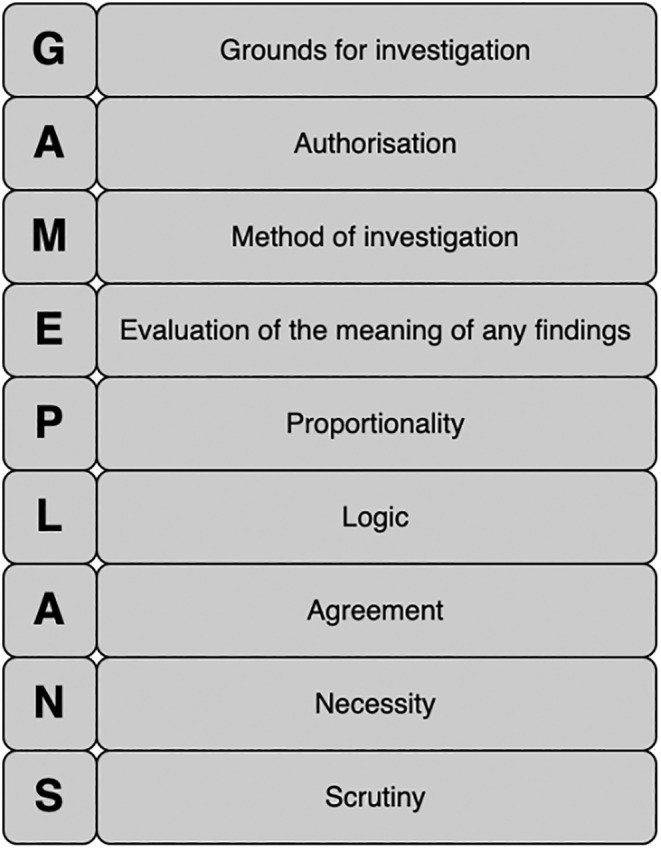
The GAMEPLANS structure.

This work aims to help investigators to form robust and effective DESs and does so through the proposal of a DES template, structured around the GAMEPLANS acrostic (see Figure 1). Nine fundamentals components required of all DESs are defined—“*G*”–*Grounds for investigation*; “*A*”–*Authorization*; “*M*”–*Method of investigation*; “*E*”–*Evaluation of the meaning of any findings*; “*P*”–*Proportionality*; “*L*”–*Logic*; “*A*”–*Agreement*; “*N*”–*Necessity*; “*S*”–*Scrutiny*. In addition to these components, each maintains a number of subtasks which an investigator must address (and evidence how they have addressed these) when forming a DES and in total, 34 subtasks are identified. The GAMEPLANS template is designed for use by police investigators and their wider investigative teams and is discussed in detail below.

### 3.1 “G”—Grounds for investigation

Many jurisdictions place legal requirements upon police which must be met when conducting an investigation. In England and Wales, the Human Rights Act 1998 places “a number of obligations on the police service which must be met in order to support the lawful interference with the rights of an individual” [22]. The College of Policing [22] states that a police investigation may be carried out following the commission of a crime, to determine if a crime has been committed, or, to explore an event where “the police believe a crime may be committed.” In all cases, those conducting any inquiries must have established an appropriate and reasonable set of grounds from which any need to investigate is based [18]. The Royal Borough of Greenwich [23] states that “reasonable grounds is what an ordinary person would think was fair if they had all the information the police officer has.” When considering reasonable grounds, much commentary regarding what is “*reasonable conduct*” focuses on the police powers of stop and search, where it is made clear that “reasonableness” is to be assessed objectively and based on the information available to an officer at that time [24], and not on any “hunch or instinct” that may be held [25]. What is clear, is that establishing and defining any reasonable grounds from which a need to conduct an investigation is based is of fundamental importance when forming a DES, and the following criteria should be noted.

1. *The proposed DES is required in order to pursue a reasonable line of inquiry* (*RLOI*) *and details of the RLOI are documented*: Horsman [26] states that “many jurisdictions will establish the legality of their law enforcement's investigative conduct through an evaluation of its reasonableness given the circumstances of any inquiry being conducted.” Consideration of the reasonableness of any potential investigative actions is required and justified, and the details of any RLOI that is intended to be pursued must be documented and described.
2. *Reliable information exists which gives rise to the need for a DES*, *and the investigative actions proposed within it* (*as part of the* “*method of investigation*”–*outlined in* “*M*”): In pursuance of their RLOI, investigators should ensure that they have information that they consider to be reliable, which invokes the requirement to investigate any digital devices/data. As a result of this information, there is a need to develop a DES. It should be made clear why any information they seek to rely upon is considered reliable, and what measures/methods they have used to determine this.
3. *Any grounds for the investigation are neither speculative nor based on conjecture*, *rather founded upon available objective information which can be evidenced*: Practitioners must be sure that they are basing their need to conduct an investigation of any digital device/data on available information/intelligence, rather than mere conjecture.
4. *The trustworthiness of all information intended to be relied upon for the purposes of informing and justifying the proposed DES has been thoroughly and appropriately evaluated*. *Information deemed dependable has been distinguished from any information which is considered questionable*: All information/intelligence used to form any reasonable grounds to justify an investigation must be evaluated in order to determine whether it can be relied upon. Where any doubt exists, such information/intelligence should be distinguished from that which is considered trustworthy and any reasons for this distinction should be documented.
5. *Appropriate investigative hypotheses have been defined for the case circumstances*, *and these are recorded*: Taking into account all available information regarding a current inquiry, any officer must appropriately formulate their investigative hypotheses which they will test as part of the investigation.

### 3.2 “A”—Authorization

Any course of investigative conduct must commence following authorization from an appropriate entity [27]. As a result, any DES must consider and address the following.

1. *Authorization for the proposed investigative actions outlined in the DES has been sought and granted by those in a position to do so*, *and that need to do so*: Consideration should be given to who must authorize any actions, or whether authorization is provided under specific regulations or standard operating procedures.
2. *A complete and accurate strategy document outlining all investigative processes and underpinning reasoning has been provided to any authorizing party*(*s*) *so that they understand the remit of any proposed investigative actions in the DES*, *and their decision to authorize it can be made correctly and in full confidenc*e: It is important to note that authorization can only be effective if those providing it are fully informed of the circumstances which they are providing approval for. This requires full details of the case and requirements to be provided accurately for assessment.
3. *All correct procedures have been followed*, *and all relevant documentation has been completed as part of seeking authorization for the DES*, *and all relevant records of the authorization processes have been retained and appropriately stored*: There are two components required here; first, those seeking authorization for a DES must do so following their organizations approved procedures to ensure any resulting approval is valid. Second, all records of the authorization must be kept in case later scrutiny of them is required.
4. *Any reasonably foreseeable legal*, *ethical and professional concerns regarding the legitimacy of any proposed conduct within the DES have been acknowledged and properly addressed*, *mitigated or managed* (*and details of how this has been done are recorded*): The authorization process must attempt to identify any wider investigative issues that may impact the validity of any investigative work taking place.

### 3.3 “M”—Method of investigation

Fundamental to all DESs are details of the appropriate investigative methods, processes, and practices that are intended for deployment and their purpose. A DES should address the following points in regard to any methods of investigation.

1. *The purpose of the investigation is defined and all proposed investigative actions regarding any digital devices/data have been defined and described in full*: Those proposing a DES should be clear as to what they are trying to achieve through their proposed deployment methods (types of data etc.) and those actions intended to be used to get this data should be outlined in full. In essence, it should be clear what is intended to be done and why.
2. *Appropriate expertise has been consulted when determining the proposed list of investigative actions*: When determining which methods of investigation are appropriate, those with the necessary relevant expertise to inform this decision should be consulted.
3. *The scope of the proposed investigative conduct has been appropriately defined*, *and it is clear and understood by all those involved in the investigation*: It is important to understand that any investigative conduct should be appropriately bounded [26] and this should be clear to all parties involved. This prevents over‐invasive investigative procedures from being deployed that add no material value to an investigation.
4. *The chosen course of investigative activity takes into account*, *and is appropriate for the type of offense alleged to have taken place*, *its severity and the known or reasonably believed circumstances surrounding it*: Any methods of investigation must be appropriate for the suspected offense being investigated and effectively address the needs of investigating such an offense. Whilst not ideal, it must be recognized that law enforcement have access to a finite set of resources, and these must be prioritized appropriately [28]. Such prioritization often takes into account the type of offense alleged and the severity of it (e.g., volume crime vs. serious crime). In an ideal world, every case would receive the maximum available amount of investigative resources, but this is not reflective of the current reality. Instead, it is suggested that a DES recognizes the circumstances of any alleged incident and sets out a course of investigative activity that is appropriate for it, given any conditions and limitations they any officer and their police force are operating under.
5. *The impact of any investigative actions upon any victim/defendants has been considered and appropriate steps have been taken to limit unnecessary intrusion*, *impact or inconvenience*: Particularly in the case of potential victims, considerations of their needs, and concerns must be given [29] and their rights set out in the Code of Practice for Victims of Crime in England and Wales 2020.

### 3.4 “E”—Evaluation of the meaning of any findings

Any DES must not just define how any device/data is going to be examined, but also how any findings are going to be evaluated throughout the course of any investigation and the impact of such evaluations. The following points must be addressed:

1. *Appropriate procedures*, *support*, *and expertise have been identified to support the process of evaluating the meaning of both any preliminary findings*, *and results generated from the completion of any analysis of devices and their data*. *Any original investigative hypotheses will be evaluated against these findings*: *Any findings must be evaluated for meaning and their impact upon any initial investigative hypotheses considered*. *This may require support from domain experts within any identified investigative team*.
2. *Appropriate milestones in the overall investigation have been defined that allow the DES's continued* “*fitness for purpose*” *to be assessed in case strategic changes need to be made*: *A DES must be dynamic* [7] *and those creating it must factor in appropriate points within an investigation where the DES can be assessed to determine whether it still describes a suitable cause of investigative conduct*.
3. *Appropriate milestones in the overall investigation have been defined that ensure any findings are reviewed in a timely manner*: *While it is acknowledged that it is important to evaluate the meaning of any findings*, *it is equally important to ensure that any findings are reviewed in a timely manner*. *Specific points in an investigation should be defined so that frequent assessments can take place*.
4. *Consideration of the potential foreseeable impact of any findings has been given and how this may determine future conduct within the investigation*: *As part of any evaluation of meaning*, *it is important to assess how any findings might impact future investigative behavior*.

### 3.5 “P”—Proportionality

The College of Policing [22] states that “actions taken during an investigation must be proportionate to the crime or incident.” Proportionality is a central concept in policing [28] requiring attention when defining investigative conduct, where the following points are noted for address:

1. *All investigative actions defined within the DES are considered proportionate to the needs of the inquiry*: Evidence should be provided that any proposed actions within a DES have been assessed for proportionality and that the conduct is considered proportionate, and why.
2. *Alternative courses of investigative action have been identified*, *documented*, *evaluated*, *and ultimately determined unsuitable*: As part of a proportionality assessment, all investigative possible conduct has been assessed and non‐proportional conduct has been justifiably discarded.
3. *When determining the proposed course of investigative action*, *all case circumstances have been considered including the severity of the alleged offense*, *risks posed to both suspects and society*, *and the potential for further harm to be caused*: An officer may reference any appropriate local and national policy that their police force is adhering to in order to identify what is considered acceptable conduct.

### 3.6 “L”—Logic

When considering the “logicalness” of any investigative conduct, it is necessary to understand any overarching investigative needs. Particularly in relation to the methods of investigation (defined in “M”), whilst it may be technically possible to “do something,” it may not be feasible given any current limitations in regards to resources or skills. When assessing logicalness as part of a DES, the following points require assessment.

1. *All investigative actions identified are logical and achievable given current resources*, *knowledge and expertise*: An appraisal of what an investigative team can do given all current operating restrictions is required. Any DES should define conduct that is technically achievable.
2. *All investigative actions identified are appropriate for addressing the all identified investigative hypotheses*: As part of an investigation the defined investigative conduct must be of a type that allows any investigative hypotheses to be addressed and evaluated.
3. *All investigative actions identified can be conducted in an acceptable time frame*: An effective investigation must also be one that is conducted within an appropriate time period. It should be evidenced that any investigative conduct can be completed within a time frame that is suitable for the needs of any investigation, taking into account victims, defendants and criminal justice system requirements.
4. *Any reasonably foreseeable risks to the success of the inquiry have been identified and steps to manage/mitigate them are documented*.

### 3.7 “A”—Agreement

It is important to distinguish “agreed” from “authorized” (noted above). Whilst authorized concerns establishing the appropriate permissions to carry out those actions in a DES, here, we are concerned with seeking agreement from with an investigative team as to what investigative actions are appropriate.

1. *The proposed course of investigative conduct has been evaluated and agreed by all involved in the investigation* (*wider investigative team*): All parties involved within an investigation have evaluated any proposed conduct within a DES and agree that it is appropriate for the case in question. This should require consultation with digital evidence specialists within the team.
2. *Any conflicts or disagreements in regard to suggested courses of investigative actions have been documented and steps taken to address these*.

### 3.8 “N”—Necessity

Similar to proportionality, necessity is a core concept considered when assessing the behavior of police. The College of Policing [24] states that “as policing professionals, we are expected to respect and protect human dignity and uphold human rights … we make decisions about human rights according to the principles of proportionality, legality, accountability, necessity, non‐discrimination, and humanity.” When considering necessity, the following points require consideration.

1. *Alternative courses of action have been evaluated*, *and the proposed strategy is considered necessary in order to effectively pursue the intended reasonable lines of inquiry outlined in* (*G*): An assessment of the risks of failing to do those actions that are being proposed within the DES should be carried out and any results should be used to justify the necessity of any proposed conduct.
2. *All proposed investigative actions can be justified and defended*.

### 3.9 “S”—Scrutiny

The maintenance of records that allow any conduct to be retrospectively assessed is fundamental good practice for investigative disciplines [30].

1. *An audit trail will be maintained*, *stored appropriately*, *and made available on request*: All investigative conduct should be documented in full, with sufficient detail to permit the scrutiny of it by a third party. Any records should be maintained in an appropriate format, allowing access to them, and stored securely.
2. *Details of all key decisions made and any justification that underpins them will be recorded*.
3. *Details of all investigative processes undertaken and their outcomes will be recorded by all parties conducting any works*.
4. *Accepted good practice in regards to contemporaneous record taking is acknowledged and understood*, *and will be followed during the course of the investigation*: Those involved in the investigation should maintain contemporaneous records of their actions in a way that aligns with accepted best practices both within their organization and their field.
5. *Any audit trail will maintain sufficient detail to allow an investigative process to be fully understood*, *evaluated*, *and repeated if required*.

## 4 GAMEPLANS IN ACTION

Whilst section 3 has outlined the components of the GAMEPLANS structure, here we will consider how it could be deployed. A DES may be considered to “wrap around” an investigation and guide it from start to finish, and therefore, the various elements of it could be invoked at timely intervals. Consider the scenario where police are provided intelligence by an online service provider of a specific user who has been uploading illegal content to their website, leading to the identification of an alleged suspect and a planned arrest, and search and seizure of their property. Following GAMEPLANS, the investigative team may ask themselves before attending a property what their grounds to investigate (G) are (arguably obvious in this case due to the supply of intelligence, however, they should evaluate its reliability) and any methods of investigation (M) they may seek to invoke which dictates their course of investigative conduct regarding any devices they may find during a search. This course of conduct should be logical (L), allow for any findings to be evaluated (E) at an appropriate period in the investigation, and have the agreement (A) of any forensic team if they are involved. Given the circumstances of their case, officers should evaluate the potential scope of the offense and determine whether their suggested strategy is proportionate (P) and necessary (N) for determining whether their alleged suspect has committed an offense. If they deem this to be the case, authorization (A) for their strategy should be sought and all relevant documentation maintained to allow for scrutinization (S) of it.

The GAMEPLANS structure is intended to provide structure to an officer's strategic thinking process and prevent the omission of critical requirements of a DES.

## 5 DISCUSSIONS AND CONCLUSIONS

The proposed GAMEPLANS structure is intended to support investigators and their teams to develop robust DESs. It consists of nine core components, which it is argued that all DESs should contain. Within these nine categories are 34 subtasks that require addressing as part of the formation of a DES, and evidence should be provided as to how these have been addressed. To support this process, Appendix S1 “DES Template.xlsx” provided as Supplemental Information contains an excel spreadsheet template that can be used by officers to work directly into when constructing a DES when adhering to the GAMEPLANS acrostic.

It is argued that the GAMEPLANS DES template provides structure to the DES creation process, helping to ensure that important elements are not overlooked or missed. This is important as some components such as gaining authority for investigative actions should not be done retrospectively. In some cases, the template may simply serve as a helpful reminder, and codify common sense elements of the DES creation process. In all cases, particularly where the provided DES template is used, a robust, transparent record of the DES should be maintained which can be evaluated by those needing to do so. Fundamentally, it is hoped that the proposed DES template helps officers to create effective and appropriate strategies which lead to thorough investigations where digital evidence is involved.

Finally, it is acknowledged that on face value, the proposed GAMEPLANS structure and its subtasks may seem burdensome; concerning at a time when police resources are already stretched, however it is argued that they are all necessary to ensure a robust DES is formed. It is hoped that over time and through consistent use, the template can be adhered to at relative speed, and in some cases, if the template prevents important investigative elements from being overlooked, this may itself save time in the long run.

## CONFLICT OF INTEREST STATEMENT

The author has no conflicts of interest to declare.

## Supporting information


Appendix S1.
